# Changes in psychological wellbeing, attitude and information-seeking behaviour among people at the epicentre of the COVID-19 pandemic: a panel survey of residents in Hubei province, China

**DOI:** 10.1017/S0950268820002009

**Published:** 2020-09-02

**Authors:** Xi Chen, Haiyan Gao, Yuchun Zou, Fen Lin

**Affiliations:** 1 Department of Media and Communication, City University of Hong Kong, Kowloon, Hong Kong; 2 Institute of Sociology, Chinese Academy of Social Sciences, Beijing, China

**Keywords:** China, COVID-19, information-seeking behaviour, perceived discrimination, psychological distress

## Abstract

While most research focuses on the clinical treatment of COVID-19, fewer studies have investigated individuals' responses towards this novel infectious disease. This study aims to report the temporal changes in individuals' psychological wellbeing, perceived discrimination, sociopolitical perceptions and information-seeking behaviours among the general public in Hubei, China. Data were obtained from a two-wave survey of 1902 respondents aged 18–80 in Hubei province during the peak and mitigation stages of the outbreak. The results showed that the prevalence of psychological distress dropped from over 75% to around 15% throughout the study period, but perceived discrimination remained stable. Female, middle-aged, well-educated respondents and those employed in government/public institutions/state-owned enterprises tended to report more distress. While respondents' attention on COVID-19 information kept high and stable, their sources of information diversified across different sociodemographic groups. Over time, people obtained more social support from neighbourhoods than from their friends and relatives or non-government organisations. Over 80% of respondents were satisfied with the performance of the central government, which was notably higher than their ratings on the local government and neighbourhood/village committees. The findings of this research are informative for formulating effective intervention strategies to tackle various psychosocial problems during COVID-19.

## Introduction

The COVID-19 pandemic has posed a huge threat to global public health and the human social fabric. Globally, a total of 5 934 936 cases and 367 166 associated deaths have been identified as of 31 May 2020 [1]. The outbreak of COVID-19 was first reported in Wuhan, the capital city of Hubei province, China in December 2019. The COVID-19 is highly contagious with confirmed cases surging to over 10 000 by the end of January 2020 in China [2]. Hubei has accounted for 96% of the deaths from the virus in mainland China so far [3]. To contain the spread of the virus, authorities imposed a lockdown on Wuhan on 23 January 2020, which triggered similar measures in 16 of its neighbouring cities in Hubei province, affecting approximately 57 million people [4]. The large-scale lockdown seemed effective at limiting the transmission of the epidemic. As some researchers estimated, without the Wuhan lockdown, the COVID-19 cases would have increased by 65% in 347 Chinese cities by the end of February 2020 [5].

Previous studies have shown that understanding psychological and behavioural responses towards emerging infectious diseases are vital for outbreak management [6, 7]. COVID-19 has caused a great psychological impact on the public [8]. The residents of Hubei may have experienced greater fear, panic and distress due to the huge numbers of infected cases and deaths coupled with the unprecedented lockdown of cities. Despite the numerous surveys that have been conducted, residents in Hubei were surprisingly underrepresented in previous surveys [9]. In this paper, we reported the temporal changes in psychological distress and associated factors, both risk factors and protective factors, during the COVID-19 pandemic.

The literature has shown several risk factors that may contribute to psychological distress during the pandemic, including fear of being infected, inadequate everyday supplies under the quarantine, and perceived discrimination [10–12]. Given that the outbreak first occurred in Wuhan, its citizens and those from surrounding areas were blamed for spreading the virus, were considered infectious, and may have been subject to discrimination and stigmatisation [13]. While specific up-to-date and accurate health information (e.g. treatment and local outbreak situation) was associated with a lower psychological impact of the outbreak [14], psychological distress can be exacerbated by ‘infodemic’ whereby people spend an inordinate amount of time on mass media that are saturated with mixed information, making it difficult for them to find trustworthy sources of information. A study found a direct association between social media exposure and anxiety during the COVID-19 pandemic [9]. Also, different venues for disseminating health information may have different impacts on psychological well-being. For example, the dissemination of health information via radio was associated with higher levels of anxiety and depression among the general population in China [15]. It suggests the government to find effective channels to increase public awareness while at the same time reduce psychological distress.

Despite various stressors during the epidemic, there are also protective factors that may improve individuals' psychological wellbeing. Prior studies have shown that social and emotional support from family and friends and social interactions can facilitate a better coping with stress, reduce negative emotions and improve mood during the COVID-19 pandemic [16, 17]. Also, satisfaction with government performance may increase compliance with preventive measures suggested by the government, thereby reducing infections [18]. However, very few studies have examined the role of neighbourhood support and evaluation of government performance in reducing psychological distress in China.

Our study may contribute to a better understanding of COVID-19-fuelled psychological distress and associated factors by using panel data of residents in Hubei. While most prior COVID-19 research used cross-sectional or repeated cross-sectional data, our advantage lies in using two-wave panel data that all of the respondents participated in both the baseline and follow-up surveys. As panel data can capture within-person changes, we may provide a more accurate description of the change of mental health status. Moreover, limited studies have focused on the psychological well-being of the general population in Hubei, China's worst-hit province by COVID-19. By surveying residents in Hubei during the peak of the outbreak and the mitigation phase of the epidemic, this study aims to examine how the general population in Hubei adapted to the COVID-19 pandemic and massive lockdown. Specifically, we reported the evolving pattern of their psychological wellbeing, perceived discrimination, as well as perceptions and behaviours throughout the COVID-19 pandemic. We also identified demographic and socioeconomic differences in how individuals respond to the pandemic. The findings of this research would be useful for designing effective intervention programmes targeting the vulnerable populations in the face of the pandemic.

## Method

### Data

The data of this study were obtained from the survey *Public attitude towards the novel coronavirus pandemic in Hubei province* conducted by the China Academy of Science and Technology Development Strategy, the Social Policy Research Institute at Renmin University, and the Institute of Sociology of the Chinese Academy of Social Sciences. The baseline survey was conducted between the 2nd and 8th of February 2020, when China was going through a phase of a rapid increase in the number of newly diagnosed COVID-19 cases and related deaths [15]. After 8 February 2020, there was a downward trend in the number of new and suspected cases. Simultaneously, the number of recovered patients showed a substantial increase during this period. The follow-up survey was conducted between 23 March and 9 April, when the daily number of newly detected cases of COVID-19 decreased to double digits in China [19]. The authorities relaxed the lockdown in Hubei on 23 March and the lockdown on Wuhan was officially lifted on 8 April 2020, after no new deaths were reported for the first time.

The survey targeted all the residents aged between 18 and 80 in the urban and rural areas of Hubei. The online survey was carried out on Epanel, a professional survey platform in China. The supplemented phone survey was conducted by trained research assistants who were university students in Hubei. Ethics approval was obtained from the ethics committee of the Chinese Academy of Social Sciences (CHN-2153, 18/0020), which conformed to the principles embodied in the Declaration of Helsinki. Informed consent from the respondents was obtained before the survey began. The baseline sample included 5239 respondents, among which 2054 were followed up in the second survey. After removing cases with missing values, the final sample included 1902 respondents.

### Measures

For the purposes of this study, we focused on four areas. First, psychological distress was measured by the degree of anxiety, fear and worry aroused by COVID-19 (1 = very low to 5 = very high). Such measures were used in other COVID-19 studies [20, 21]. The responses were dichotomised as ‘very high/high’ *vs.* ‘very low/low/neutral’. Second, perceived discrimination was measured by asking the respondents whether they had encountered discrimination because of the COVID-19 pandemic. Previous studies have used a similar single item to assess perceived discrimination because of COVID-19 [21]. Third, various sociopolitical perceptions were measured, including the anticipated time to control the epidemic (within 6 months *vs.* more than 6 months); the attitude towards the lockdown (ease the lockdown as soon as possible *vs.* not lifting the lockdown until the epidemic was eliminated); evaluation of the government's performance in controlling the epidemic and social support from relatives and friends, their neighbourhood, as well as NGOs (whether the respondent had received support with medical supplies, daily household supplies, help with children and caring for the elderly or reassurance from the above three sources). Finally, information-seeking behaviour was assessed by the attention the respondents paid to different information contents, such as statistics of infection, local necessity supplies, personal prevention strategies, government responses to the epidemic, criticisms and suggestions of disease containment work, and medical and scientific advances during the study period (six-item scale, *α* = 0.88). Similar items were used in other COVID-19 studies [22].

### Data analysis

The distribution of responses was tabulated for the baseline and the follow-up surveys. Differences across the two surveys were tested using the *t* or *χ*^2^ test. We then conducted the analysis stratified by age (⩽25, 26–45 and ⩾46), gender (male *vs.* female), education (high school or below *vs.* college or above), and occupation (employed in the government/public institutions/state-owned enterprises (SOEs) *vs.* employed in private enterprises/individual business/other). Such analyses showed the sociodemographic variations in the responses. Stata 14.2 was used to analyse the data and *P* values <0.05 were considered significant.

## Results

### Background characteristics

Table 1 displays the background characteristics of the respondents. Most respondents were men (56.11%), aged below 35 (62.77%), with a college education or above (56.84%), had a monthly income below RMB4000 (approximately US$845, 59.02%), worked in private enterprises or individual businesses (53.20%), were local non-migrant residents (84.4%), and lived in urban areas (89.38%). About 15% of the respondents were members of the Communist Party of China (CPC).

**Table 1.** Background characteristics of the respondents (*N* = 1902)

Variable	%
Age (mean/s.d.)	(33.48/12.49)
⩽25	30.49
26–35	32.28
36–45	21.61
46–55	10.15
>56	5.47
Gender	
Male	56.11
Female	43.89
Education	
Middle school or below	16.38
High school	26.78
College or above	56.84
Party membership	15.72
Occupation	
Government/public institution/SOE staff	32.88
Private enterprise staff or individual business	53.20
No work or refuse to disclose	13.82
Monthly income	
No income	5.86
⩽2000	19.69
2001–4000	33.47
4001–6000	20.70
6001–8000	11.25
⩾8001	7.44
Do not know/refuse to disclose	1.58
Migrant status	
Migrants	15.60
Non-migrants	84.40
Area	
Urban	89.38
Rural	10.62

### Psychological distress

Table 2 illustrates the changes in psychological responses, sociopolitical perceptions and information-seeking behaviour related to COVID-19 between the two waves. The results of the baseline survey showed a high prevalence of psychological distress: about 75%, 85% and 87% of the respondents felt anxious, worried and fearful about COVID-19, respectively. However, psychological distress declined significantly in the mitigation phase. In the follow-up survey, <15% of the respondents reported anxious, worried and fearful about the pandemic.

**Table 2.** Responses toward COVID-19 between the two waves

Variable	Wave 1	Wave 2	*χ*^2^/*t* test
Anxiety	75.11	12.72	49.77***
Worries	84.55	15.19	59.31***
Fear	86.96	10.88	72.19***
Perceived discrimination	44.34	42.69	1.02
*Information-seeking behaviour*			
Pay close attention to suspected, confirmed and death cases	97.99	95.48	4.37***
Pay close attention to information on necessity supplies	91.95	94.58	−3.24**
Pay close attention to personal prevention strategies	98.20	94.37	6.25***
Pay close attention to government responses to the epidemic	97.25	93.90	5.02***
Pay close attention to criticisms and suggestions of disease containment work	93.22	91.06	2.47***
Pay close attention to medical and scientific advances	97.30	93.32	5.81***
*Optimism*			
Anticipate to control the epidemic within 6 months	11.40	21.84	−8.56***
Attitude towards the lockdown			
Ease the lockdown as soon as possible	28.81	33.63	9.24**
Ease the lockdown only when the outbreak is eliminated	71.19	66.37	
*Social support*			
Community support	37.98	64.67	−16.92***
Relative/friend support	32.85	53.45	−12.98***
NGOs/volunteer groups support	14.53	34.22	−14.37***
*Satisfaction with government performance*			
Satisfaction with the central government	89.26	82.12	6.29***
Satisfaction with the local government	58.82	75.76	−11.29***
Satisfaction with neighbourhood/rural committees	49.87	75.03	−16.56***

*P < 0.05; **P < 0.01; ***P < 0.001.

As shown in Tables 3 and 4, the survey statistics stratified by sociodemographic variables suggested that the male respondents aged 26–45, with a college education or above, and working in government, public institutions, or SOEs reported significantly higher levels of psychological distress in the baseline survey. Such age, gender and occupational disparities in psychological distress disappeared in the follow-up survey. It is worth noting that although respondents with higher education were more distressed in the baseline survey, they tended to report significantly less distress in the follow-up survey.

**Table 3.** Responses toward COVID-19 stratified by age and gender

	Wave 1				Wave 2				Wave 1			Wave 2		
	⩽25	26–45	⩾46	*χ*^2^ test	⩽25	26–45	⩾46	*χ*^2^ test	Male	Female	*t*/*χ*^2^ test	Male	Female	*t*/*χ*^2^ test
Anxiety	74.48	78.21	65.66	19.57***	13.97	12.39	11.45	1.34	75.12	75.39	−0.14	12.16	13.13	−0.63
Worries	81.31	88.15	78.45	23.21***	16.03	14.73	15.15	0.49	81.40	88.88	−4.50***	14.70	15.66	−0.58
Fear	86.46	88.57	82.43	7.81*	13.10	9.66	10.77	4.54	85.19	89.58	−2.82**	11.59	10.12	1.02
Perceived discrimination	51.57	42.94	35.14	23.12***	47.76	41.66	36.36	11.39**	49.95	37.21	5.56***	44.20	40.60	1.57
*Information-seeking behaviour*														
Pay close attention to suspected, confirmed and death cases	98.10	99.12	93.94	31.43***	95.86	96.10	92.59	6.83*	98.30	97.94	0.56	95.48	95.54	−0.07
Pay close attention to information on necessity supplies	91.87	93.71	86.01	18.27***	92.93	95.80	93.60	6.64*	91.36	92.62	−0.99	94.53	94.58	−0.04
Pay close attention to personal prevention strategies	98.44	99.31	93.90	38.15***	94.83	94.63	92.59	2.13	97.91	98.54	−1.02	94.44	94.58	−0.13
Pay close attention to government responses to the epidemic	97.05	98.23	94.24	13.76**	93.62	94.15	93.60	0.23	97.34	97.45	−0.15	94.16	93.73	0.38
Pay close attention to criticisms and suggestions of disease containment work	93.76	93.80	90.14	5.24	90.69	92.49	86.87	9.08*	93.43	93.24	0.17	90.95	91.45	−0.38
Pay close attention to medical and scientific advances	97.24	98.82	92.18	38.35***	92.07	94.83	90.57	8.80*	97.63	96.98	0.88	93.50	93.13	0.32
*Optimism*														
Anticipate to control the epidemic within 6 months	13.81	8.86	15.52	14.55**	23.48	20.79	22.34	1.50	13.03	9.46	2.40*	22.03	21.40	0.32
*Attitude towards the lockdown*														
Ease the lockdown as soon as possible	37.98	25.83	21.75	32.45***	37.80	32.77	25.29	9.98**	34.84	20.81	41.19***	35.65	30.81	4.25*
Ease the lockdown only when the outbreak is eliminated	62.02	74,17	78.25		62.20	67.23	74.71		65.16	79.19		64.35	69.19	
*Social support*														
Community support	37.96	39.19	33.90	2.68	62.83	65.29	66.10	1.28	38.68	37.17	0.66	65.11	64.24	0.39
Relative/friend support	28.47	32.22	43.49	20.12***	52.71	53.49	54.79	0.34	29.37	37.67	−3.77***	52.49	55.03	−1.10
NGOs/volunteer groups support	13.36	16.67	9.59	10.01**	35.95	36.87	21.58	24.68***	15.16	13.77	0.84	36.52	31.27	2.38*
*Satisfaction with government performance*														
Satisfaction with the central government	84.67	91.59	90.17	18.61***	76.21	84.00	87.21	21.51***	87.80	91.35	−2.48	81.06	83.73	−1.51
Satisfaction with the local government	53.40	59.90	65.65	13.09**	71.38	77.85	77.10	8.80*	59.33	58.03	0.57	75.68	76.39	−0.35
Satisfaction with neighbourhood/rural committees	43.67	51.43	56.61	15.21***	71.72	76.68	75.76	4.96	49.24	50.79	−0.67	75.12	75.18	−0.03

*P < 0.05; **P < 0.01; ***P < 0.001.

**Table 4.** Responses toward COVID-19 stratified by education and occupation

	Wave 1			Wave 2			Wave 1			Wave 2		
	⩽High school	⩾College	*t*/*χ*^2^ test	⩽High school	⩾College	*t*/*χ*^2^ test	Government/SOE	Other	*t*/*χ*^2^ test	Government/SOE	Other	*t*/*χ*^2^ test
Anxiety	71.15	78.16	−3.49***	14.74	10.82	2.55*	81.03	74.25	2.95**	10.24	13.50	−1.83
Worries	81.38	86.93	−3.31**	16.34	14.37	1.18	86.81	85.20	0.85	13.58	15.72	−1.10
Fear	85.98	87.73	−1.12	13.76	8.86	3.38***	90.14	86.12	2.24*	10.04	11.76	−1.01
Perceived discrimination	42.57	45.85	−1.41	43.73	41.79	0.85	48.11	42.13	2.22*	38.98	44.46	−2.05*
*Information-seeking behaviour*												
Pay close attention to suspected, confirmed and death cases	96.67	98.97	−3.53***	94.72	95.99	−1.31	98.81	98.16	0.95	96.85	94.70	1.90
Pay close attention to information on necessity supplies	90.57	92.89	−1.82	93.73	95.15	−1.34	93.06	91.95	0.76	95.08	94.99	0.08
Pay close attention to personal prevention strategies	97.52	98.69	−1.87	92.51	95.71	−2.98**	98.61	98.54	0.10	95.28	94.02	1.01
Pay close attention to government responses to the epidemic	95.91	98.22	−3.02**	93.12	94.50	−1.24	97.62	97.67	−0.06	94.29	94.41	−0.09
Pay close attention to criticisms and suggestions of disease containment work	92.81	93.62	−0.69	89.31	92.35	−2.29**	92.86	94.28	−1.08	91.93	90.84	0.71
Pay close attention to medical and scientific advances	95.91	98.31	−3.18**	92.14	94.22	−1.79	98.61	97.48	1.44	94.88	93.35	1.18
*Optimism*												
Anticipate to control the epidemic within six months	11.24	9.18	3.26**	23.40	20.80	1.31	8.80	11.13	−1.40	23.18	20.00	1.39
*Attitude towards the lockdown*												
Ease the lockdown as soon as possible	33.20	25.53	12.18***	36.76	31.54	4.78*	33.85	25.16	11.56**	34.13	32.85	0.22
Ease the lockdown only when the outbreak is eliminated	66.80	74.47		63.24	68.46		66.15	74.84		65.87	67.15	
*Social support*												
Community support	34.58	40.68	−2.68**	60.79	67.86	−3.17**	37.30	39.74	−0.91	68.65	64.27	1.70
Relative/friend support	32.34	33.43	−0.49	47.89	57.68	−4.22***	30.53	33.76	−1.25	52.58	53.59	−0.37
NGOs/volunteer groups support	13.56	15.27	−1.03	31.76	36.00	−1.92	15.98	14.82	0.59	36.11	32.91	1.24
*Satisfaction with government performance*												
Satisfaction with the central government	87.55	90.61	−2.12*	79.24	84.14	−2.75**	90.85	89.29	0.95	82.28	82.45	−0.08
Satisfaction with the local government	62.59	55.99	2.88**	74.94	76.12	−0.59	59.01	58.34	0.25	79.13	75.12	1.75
Satisfaction with neighbourhood/rural committees	48.89	50.89	−0.86	72.85	76.68	−1.90	49.01	50.29	−0.47	76.38	74.93	0.62

*P < 0.05; **P < 0.01; ***P < 0.001.

A closer look at the within-person change in psychological wellbeing revealed that more than 80% of the respondents reported lower levels of anxiety, worries and fear in the follow-up survey (Appendix 1). Interestingly, respondents with a college education or above reported significantly lower levels of psychological distress in the follow-up survey than those with high school education or below (85.35 *vs.* 79.03, *P* < 0.001). There were no age, gender or occupational differences in the change of psychological wellbeing between the two waves.

### Perceived discrimination

Of the participants, about 45% in the baseline survey felt being discriminated against since the outbreak of the pandemic. The prevalence of perceived discrimination remained largely the same (43%) in the follow-up survey. The youngest generation reported most perceived discrimination: about 50% of respondents aged below 25 reported being discriminated against in both waves. More male respondents perceived being discriminated against than females in the baseline survey (50% *vs.* 37%), but the gender difference in perceived discrimination became insignificant in wave 2. While respondents who worked in government, public institutions or SOEs were more likely to perceive being discriminated against than their counterparts who worked in private sectors or had no work in wave 1 (48% *vs.* 42%), they reported a lower percentage of perceived discrimination than their counterparts in wave 2 (39% *vs.* 44%). There was no educational difference in perceived discrimination in both waves.

### Information-seeking behaviour

The results suggested that more than 90% of respondents paid close or some attention to information regarding COVID-19 in both waves. Older and less-educated respondents paid relatively less attention to the COVID-19 information in both waves. There were no gender or occupational differences in information-seeking behaviour.

Further analysis on sources of information (only available in wave 1, Appendix 2) suggested the sources of information diversified across different sociodemographic groups. Specifically, elder respondents preferred to get news from traditional sources, such as TV/radio/newspaper or friends and relatives. They were less likely to consume information from newly emerging media forms, including Weibo, short video Apps and knowledge sharing websites. Female respondents favoured TV/radio/newspaper over social media platforms (e.g. Weibo, QQ and knowledge sharing websites). Compared to highly-educated respondents, those with high school education or below were less likely to get information from social media websites.

### Expected time to control the epidemic

Over time, people were more optimistic about the length of the outbreak. While only 11.4% of respondents thought that the epidemic would be controlled within 6 months in the baseline survey, this percentage nearly doubled (21.84%) in the follow-up survey. In the baseline survey, people aged over 65 (15.52%), males (13.03%) and people with lower education (11.24%) were more likely to believe that the epidemic would be controlled within 6 months. There were no age, gender, educational or occupational differences in perceived time to control the epidemic in wave 2.

### Attitude towards the lift of the lockdown

Consistent with the rising optimistic attitude towards controlling the epidemic, more people supported to lift the lockdown as soon as possible across the study period (29% in wave 1; 34% in wave 2). However, the majority (over 65%) of respondents still thought it was better to lift the lockdown only after the epidemic was eliminated. Further analyses showed that male respondents, aged below 25, with lower education, and working in government, public institutions or SOEs were more likely to support to lift the lockdown as soon as possible in both waves.

### Evaluation of government performance

In wave 1, nearly nine-in-ten respondents (89.26%) rated the performance of the central government in controlling the pandemic as excellent or good. In contrast, only about 59% and 50% of respondents said the same about the performance of the local government and neighbourhood/rural committees. While the satisfaction with the central government declined slightly in wave 2 (82.12%), the satisfaction with the local government and neighbourhood/village committees increased significantly to 76% and 75%, respectively. Moreover, the results in Table 4 showed that older people were more satisfied with the performance of the central and local governments in both waves. Interestingly, people with higher education tended to report higher satisfaction with the central government (90.61% *vs.* 87.55% in wave 1, *P* < 0.05; 84.14% *vs.* 79.24% in wave 2, *P* < 0.01), but lower satisfaction with the local government in wave 1 (55.99% *vs.* 62.59%, *P* < 0.01). There were no gender or occupational differences in the evaluation of government performance at different levels.

### Social support

As for the three sources of social support, the respondents were most likely to receive neighbourhood support (38% in wave 1; 65% in wave 2; *P* < 0.001), and least likely to obtain support from NGOs or volunteer groups (15% in wave 1; 34% in wave 2; *P* < 0.001), with support from relatives or friends (33% in wave 1; 53% in wave 2; *P* < 0.001) in between. All of the three types of social support increased over time. Analysis stratified by sociodemographic variables indicated that people aged over 46 got the most support from relatives and friends (43.5% in wave 1; 54.8% in wave 2), but received the least support from NGOs or volunteer groups (9.6% in wave 1; 21.6% in wave 2). Female respondents received more support from relatives and friends than males. However, a higher percentage of male respondents got support from NGOs/volunteer groups (36.5%) in wave 2. Respondents with a higher education received more support from neighbourhoods and friends/relatives in wave 2. There were no occupational differences in social support obtained.

## Discussion and conclusion

The COVID-19 epidemic was first identified in Wuhan, the capital of Hubei province in China. Although stringent public health measures are successful in disease containment, there is little research on the extent to which the disruptions affected the psychological wellbeing of residents exposed to such an unprecedented lockdown. By using a two-wave survey of 1902 respondents in Hubei province, China, this study is among the first to analyse the longitudinal changes in individual responses throughout the pandemic. The findings yielded several important implications.

First, the prevalence of psychological distress among Hubei residents in the peak of the outbreak (i.e. wave 1) seemed higher than prior studies using national samples [23]. Female, middle-aged, well-educated respondents and those employed in government/public institutions/SOEs tended to report more distress. Such findings were consistent with previous studies showing that women are more vulnerable to stress and more likely to develop post-traumatic stress disorder [24]. People with higher education tended to be more concerned about the epidemic, probably because of their high self-awareness of health [25]. Also, middle-aged people may bear more stress as they need to take care of their children and ageing parents during the epidemic. Furthermore, government or state enterprises' employees were more distressed, partly because they were pushed to the frontline of the community battle against COVID-19. They may have been more worried due to the high levels of risk exposure to the COVID-19 virus. Health authorities may target these vulnerable groups for early psychological interventions. In addition to behavioural therapy that could reduce anxiety through relaxation techniques and changes of routine activities, psychological intervention programmes, such as cognitive behavioural therapy and mindfulness-based cognitive therapy, may also help mitigate maladaptive coping behaviours and alleviate stress in people [26].

The results also revealed a greater decrease in psychological distress among respondents with a college education. It may be that well-educated people have more access to the updated information and rational judgement about the epidemic, thereby adjusting their risk perceptions [27, 28]. To improve the psychological health of people with lower education, the government should spread simple and easy-to-understand messages that facilitate an objective understanding of the epidemic and reduce unnecessary panic and stress.

Second, despite the great reduction in psychological distress, the percentage of respondents reporting perceived discrimination remained stable. Since discrimination and stigma may isolate people and prevent them from seeking medical help [29], effective behavioural and health education interventions are needed to encourage the appropriate health-seeking behaviours of the infected population. Also, public health officials should deliver rapid and clear messages effectively to the entire population because an accurate understanding of the epidemic can reduce stigmatising attitudes in the general public [30]. Considering that stigma towards the affected population of infectious diseases could continue even after containment of the outbreak [31], it requires a long-term, sustained commitment to reducing stigma and discrimination.

Third, the COVID-19 crisis highlights the need for authentic, accurate and up-to-date information. There is a high demand for information during the pandemic as over 90% of respondents in our sample paid attention to COVID-19-related information (e.g. statistics of infection, personal prevention strategies, etc.), which is consistent with recent studies among other populations. For example, a study of the Vietnamese population found that their most requested information was the latest updated news on the epidemic, followed by information about disease symptoms and updated news on the outbreak [22]. While reliable information from governments and health authorities helps people make informed decisions, consuming excessive or inaccurate information may lead to an ‘infodemic’ that exacerbates anxiety and fear towards the pandemic [9]. People may feel afraid and anxious by the constantly changing alerts and sensationalised news headlines and images regarding the rapid spread of the virus. Such information-induced anxiety was reduced when established medical experts, such as Zhong Nanshan, Li Lanjuan and Zhang Wenhong clarified the epidemic situation to dispel rumours and raise public awareness to comply with preventive measures. It is thus important to disseminate scientific knowledge about emerging infectious diseases to increase compliance and ease public panic [32]. Also, individuals should carry out ‘information diet' by controlling the extent and type of information they consume. As the WHO suggests, people should seek good-quality and accurate information from trusted sources, such as the WHO website and local health authority platforms, once or twice preferably at specific times during the day [33].

Fourth, neighbourhoods play an important role in providing social support needed for the battle against the COVID-19 crisis. As the grass-root arm of the government, neighbourhood committees in China provide the institutional infrastructure for limiting and monitoring movement, conducting regular door-to-door temperature checks, and supplying groceries and other necessities to residents under quarantine. Our results suggested that females, older people and less-educated people received less support from neighbourhoods. Stakeholders and health policy-makers should collaborate to provide instrumental and emotional support to affected populations, especially seniors and vulnerable groups.

Finally, despite an increase in the satisfaction with the local government and neighbourhood/village committees, respondents kept reporting a higher satisfaction with the performance of the central government. The dissatisfaction with lower levels of government at the early stage of the outbreak may be due to their mismanagement of the outbreak, including delayed response and information suppression [34]. The rising satisfaction rates may be attributed to a series of effective measures taken by the local government and neighbourhood/village committees [35]. Since a lack of trust in government institutions may decrease compliance with recommended health practices and undermine the effectiveness of public health measures [36], it is important to raise the public trust and satisfaction with the government.

This study has several limitations. First, the survey was mainly conducted online, which could induce some biases, such as a low representation of the elderly, rural population and those with limited access to the Internet. However, we have supplemented the online survey with a telephone survey to sample non-netizens. Second, the self-reported anxiety and fear may not be aligned with the objective assessment made by mental health professionals. Nevertheless, psychological impact and stress are more based on personal feelings, and the self-reporting method is widely used during the COVID-19 pandemic [37]. Third, since not many studies have examined the role of neighbourhood support and satisfaction with government performance in reducing distress during the pandemic in China, this study contributes to the existing discussion by focusing on social support and satisfaction with government performance that may lower individuals' psychological distress. However, as some studies have shown, other protective factors, such as confidence in doctors, personal precautionary measures and good ventilation and concern from the company may also reduce distress [14, 15, 38]. Thus, a more thorough examination in the future studies can further explore how the role of neighbourhood support and institutional satisfaction interact with these other protective factors in shaping the COVID-19-fuelled psychological distress. Fourth, the items for measuring the nonspecific psychological distress were not validated although there were adapted from previous studies and the internal consistency was satisfactory [20]. Our analysis results were largely consistent when we compared the three items with other standardised scales of psychological distress. In our follow-up survey (wave 2) we included the Chinese Health Questionnaire-12 items scale (CHQ-12) which has been validated in the general population in mainland China [39]. Since this study aims to use the panel data to address within-person change, we decided to use these three items of psychological distress measurements that appeared in both the baseline and follow-up surveys. In addition, other measures such as sociopolitical perceptions were designed for the context of COVID-19 and were not validated before. Future studies may further test the validity of these variables among the Chinese population.

Despite the limitations, this study was among the first to report the changes in psychological wellbeing, perceived discrimination and psychosocial perceptions and behaviours during the COVID-19 pandemic among residents in Hubei province, China. The findings of this research are informative for formulating effective intervention strategies to tackle various psychosocial problems during COVID-19. Also, understanding China's experiences in coping with COVID-19 would be useful for other countries to deal with the challenges.

## Data Availability

The data that support the findings of this study are available from The Chinese Academy of Social Sciences. Restrictions apply to the availability of these data, which were used under licence for this study. Data are available from the authors with the permission of The Chinese Academy of Social Sciences.

## Appendix

**Appendix 1.** Within-person change of psychological distress between the two waves

	Full sample	Age				Gender			Education			Occupation		
		⩽25	26–45	⩾46	*χ*^2^ test	Male	Female	*χ*^2^ test	High school or below	⩾College	*χ*^2^ test	Government/public institution/SOE	Other	*χ*^2^ test
Anxiety														
Lower	82.81	81.72	84.49	79.12	7.08	83.03	82.77	3.73	79.03	85.35	12.85***	85.83	81.77	4.39
Same	11.46	11.90	10.83	12.79		12.16	10.60		12.65	10.35		9.06	12.44	
Stronger	5.73	6.38	4.68	8.08		4.81	6.63		7.62	4.29		5.12	5.79	
Worry														
Lower	84.86	83.79	85.95	83.16	5.27	84.73	85.54	1.79	82.92	86.29	4.39	87.01	85.25	1.69
Same	10.67	10.34	10.44	12.12		10.27	10.72		11.79	9.89		9.65	10.03	
Stronger	4.47	5.86	3.61	4.71		5.00	3.73		5.28	3.82		3.35	4.73	
Fear														
Lower	90.22	87.76	92.00	88.89	12.77*	89.82	90.84	0.75	88.33	91.70	6.01*	90.55	89.87	2.59
Same	6.99	8.79	6.15	6.40		7.35	6.51		8.23	5.97		7.48	6.75	
Stronger	2.79	3.45	1.85	4.71		2.83	2.65		3.44	2.33		1.97	3.38	

**P* < 0.05; ***P* < 0.01; ****P* < 0.001.

## Appendix

**Appendix 2.** Sources of epidemic-related information (only available in wave 1)

Sources of information	Full sample	Age				Gender			Education			Occupation		
		⩽25	26–45	⩾46	*χ*^2^ test	Male	Female	*t* test	High school or below	⩾College	*t* test	Government/public institution/SOE	Other	*t* test
TV/radio/newspapers	61.15	59.76	60.24	67.00	5.11	58.88	64.17	−2.34*	63.55	59.42	1.82	61.74	60.04	0.64
Community radio/high-pitched speakers	25.20	22.63	25.76	28.28	3.70	24.20	26.66	−1.22	27.59	23.41	2.07*	23.67	27.32	−1.53
Friends and relatives	22.98	21.93	19.49	37.04	40.53***	23.63	22.20	0.73	23.40	22.57	0.42	19.33	22.78	−1.55
News websites/Apps	60.25	62.52	62.98	46.46	27.99***	61.44	59.23	0.97	52.09	66.42	−6.36***	62.33	59.56	1.05
WeChat	61.36	58.72	64.54	55.56	10.29**	61.44	61.28	0.07	58.87	63.25	−1.93	62.33	63.51	−0.45
Weibo	39.17	51.81	38.49	16.84	101.23***	41.49	36.43	2.24*	30.17	45.99	−7.05***	41.03	36.00	1.91
QQ	22.35	35.58	18.02	11.45	89.74***	25.99	17.61	4.36***	22.04	22.57	−0.27	24.06	18.73	2.44*
Short video Apps	36.90	43.18	36.92	24.58	29.16***	36.67	37.15	−0.21	38.79	35.54	1.45	33.33	38.13	−1.83
Knowledge sharing websites	16.24	22.11	15.38	7.74	30.98***	17.84	14.23	2.12*	14.29	17.72	−2.00*	17.55	14.48	1.57
Moment	57.43	53.44	58.53	62.64	5.34	59.52	54.79	1.78	57.85	57.30	0.20	59.64	59.01	0.21

**P* < 0.05; ***P* < 0.01; ****P* < 0.001.
